# Child health nurses in the Solomon Islands: lessons for the Pacific and other developing countries

**DOI:** 10.1186/1478-4491-10-45

**Published:** 2012-11-21

**Authors:** Samantha Colquhoun, Divi Ogaoga, Mathias Tamou, Titus Nasi, Rami Subhi, Trevor Duke

**Affiliations:** 1 Centre for International Child Health, University of Melbourne, Royal Children's Hospital, MCRI,, 50 Flemington Road, Parkville,, 3052, Melbourne, Australia; 2 Ministry of Health, Solomon Islands; 3 School of Medicine and Health Sciences, University of Papua New Guinea, Papua New Guinea

**Keywords:** Solomon Islands, Child health, Nurses, Developing countries, Pacific Islands

## Abstract

**Objectives:**

To understand the roles of nurses with advanced training in paediatrics in the Solomon Islands, and the importance of these roles to child health. To understand how adequately equipped child health nurses feel for these roles, to identify the training needs, difficulties and future opportunities.

**Design:**

Semi-structured interviews.

**Settings:**

Tertiary hospital, district hospitals and health clinics in the Solomon Islands.

**Participants:**

Twenty-one paediatric nurses were interviewed out of a total of 27 in the country.

**Results:**

All nurses were currently employed in teaching, clinical or management areas. At least one or two nurses were working in each of 7 of the 9 provinces; in the two smaller provinces there were none. Many nurses were sole practitioners in remote locations without back-up from doctors or other experienced nurses; all had additional administrative or public health duties. Different types of courses were identified: a residential diploma through the University of Papua New Guinea or New Zealand and a diploma by correspondence through the University of Sydney.

**Conclusions:**

Child health nurses in the Solomon Islands fulfill vital clinical, public health, teaching and administrative roles. Currently they are too few in number, and this is a limiting factor for improving the quality of child health services in that country. Current methods of training require overseas travel, or are expensive, or lack relevance, or remove nurses from their work-places and families for prolonged periods of time. A local post-basic child health nursing course is urgently needed, and models exist to achieve this.

## Introduction

With the final push to achieve the Millennium Development Goals (MDG), including that to reduce child mortality rates, there is increasing recognition of the need for strong health systems and comprehensive child health programmes in the poorest countries. This will be just as relevant *beyond* the MDG target date of 2015. Advances in prevention and treatment of childhood illnesses, responses to emerging diseases and public health threats, more detailed understanding of causes of morbidity and child deaths, and an increased focus on neonatal health have increased the complexity of child health since the decade before the Millennium Goals were declared [1]. In many countries this increasing technical and programme complexity has not been matched with advances in providing a sustainable workforce that has the knowledge and skills to support these advances [2].

The Solomon Islands child mortality rate has been steadily falling since 1990, now estimated by the World Health Organization (WHO) to be 27 per 1000 live births [3]. Seventy five percent of child deaths in the Solomon Islands occur in the first year of life [4]. In 2012 the country has an estimated population of 542,000, with more than 40% aged 15 years or less [5]. The most common causes of childhood deaths are pneumonia, malaria, acute gastroenteritis, and meningitis, commonly complicated by malnutrition. Among neonates the commonest causes of death are complications of prematurity and low birth weight, sepsis (skin infection, pneumonia, bacteraemia, ophthalmitis and cord infection) and birth asphyxia. Other important childhood conditions include anaemia, tuberculosis, worm infestation, rheumatic heart disease, trauma, and bone and joint infections [4,6].

Access to medical care is difficult in the Solomon Islands, because of long distances and dispersed islands, with 70% of the population living in remote or very remote areas. Most seriously ill children present to provincial hospitals, area health centres and clinics and need to be cared for in their home provinces, rather than risk the journey to the National Referral Hospital (NRH) in Honiara by boat or small plane.

In the Pacific, the doctor/population ratio is among the lowest in the world. In the Solomon Islands and Papua New Guinea (PNG) the ratios are 11 and 7 per 100,000 population respectively [7], among the lowest in Asia and the Pacific [8]. By contrast in Australia and New Zealand there are 250 doctors per 100,000 people in the population [7]. In Pacific countries nurses predominantly provide front-line management of sick children, manage children’s wards, train junior staff, and coordinate provincial and district public health and Maternal Child Health (MCH) programmes. This is done in settings where supervision and input from medical staff is sparse or unavailable. With the current scope of child health it is unlikely that undergraduate nursing courses can equip nurses to adequately deal with these more complex roles and demands.

We sought to understand the contribution of child health nurses to the Solomon Islands health system. We explored their roles, the nurses’ perceptions of the appropriateness of their training to these roles, including whether they felt equipped to address the complexity of new child health and public health challenges, and their continuing professional development needs. We reviewed the health system and human resource context in which these nurses worked.

## Methods

Nurses who had undertaken any advanced training in child health were identified by the Child and Reproductive Health Unit of the Ministry of Health in Honiara. Data were obtained on each of the nurses, including their current position and location of employment. The nurses were contacted by radio or in person, and consent to be interviewed was sought. Semi-structured interviews were undertaken to obtain information across an number of key areas, which included the course undertaken and course components; a description of any other training received in paediatrics; the nurses’ current roles and support provided for them; the perceived limitations and difficulties in undertaking these roles and identification of additional duties such as teaching, data collection and reporting or involvement in research. The interviews consisted of 10 main questions. The questions were designed to obtain information regarding the main themes, with prompts to allow the nurses to discuss their experiences at further length. At the end of the interview nurses were asked to add any additional thoughts or comments that they considered relevant for the interviewers to know.

All nurses were interviewed in English with two interviewers present, one of whom was also fluent in Solomon Islands Pidgin. Interviews were done where possible at the nurse’s place of work. All nurses gave their permission to be interviewed. Interviews were recorded on a portable dictaphone and then transcribed by the researchers who undertook the interviews. Additional interviews and discussions to obtain background data were done with medical directors at two provincial hospitals, the head of the Ministry of Health Child and Reproductive Health unit, a paediatrician from Honiara, and a junior doctor at a provincial hospital.

Data were reviewed by exploring each theme, identifying common sub-themes and categorising comments. Recorded interviews were stored in MP3 files, transcribed and imported into NVivo 9 software (QSR International 2011: http://www.qsrinternational.com/) to undertake analysis. The project was approved by the Solomon Islands Ministry of Health Ethics Committee. All information provided was kept confidential and de-identified.

## Results

### Description of Solomon Island nurses interviewed

We identified 27 nurses who had completed a post-basic paediatrics course, and interviewed 21 of them (Table 1). The majority trained at the University of Papua New Guinea (UPNG) in Port Moresby. Of the fifteen nurses who had completed the UPNG course, six had completed a paediatrics course, which ran prior to 2004, and nine had subsequently completed the combined paediatrics and midwifery course. Twelve nurses had recently completed the distance learning course through the University of Sydney, including three nurses who had graduated from the UPNG residential course. Three other nurses had completed other residential courses in Auckland or at the Adventist University in PNG.

**Table 1:** Paediatric courses completed by Solomon Islands nurses 1990 to 2011

**Course (range of years nurses completed)**	**Number**	**Number interviewed**
Advanced diploma in Paediatrics UPNG (1990-2003)	6	6
Advanced diploma in Paediatrics & midwifery UPNG (2004-05)	9	8
Advanced diploma in Paediatrics University of Auckland (1999)	1	1
Advanced diploma in Paediatrics Seventh Day Adventist University PNG (now discontinued) (2006)	2	1
IPPC, distance education course University of Sydney (2009-10)	12	5
Total	27 ^a^	21

^a^Three of the twelve nurses who completed the distance education course had also previously completed the UPNG course and are included above in the UPNG course numbers.

One nurse was interviewed by two-way radio and the other interviews were face-to-face. Of the two UPNG-trained nurses not interviewed, one was working in a senior management position at the NRH in Honiara and was unavailable during the period of the study, and the other was working as a sole practitioner in a remote clinic in Makira Province. At the time of the study the two-way radio at this clinic was not functioning. Four nurses who had completed the distance course were not interviewed; all were working at the NRH in Honiara.

**Figure 1:**
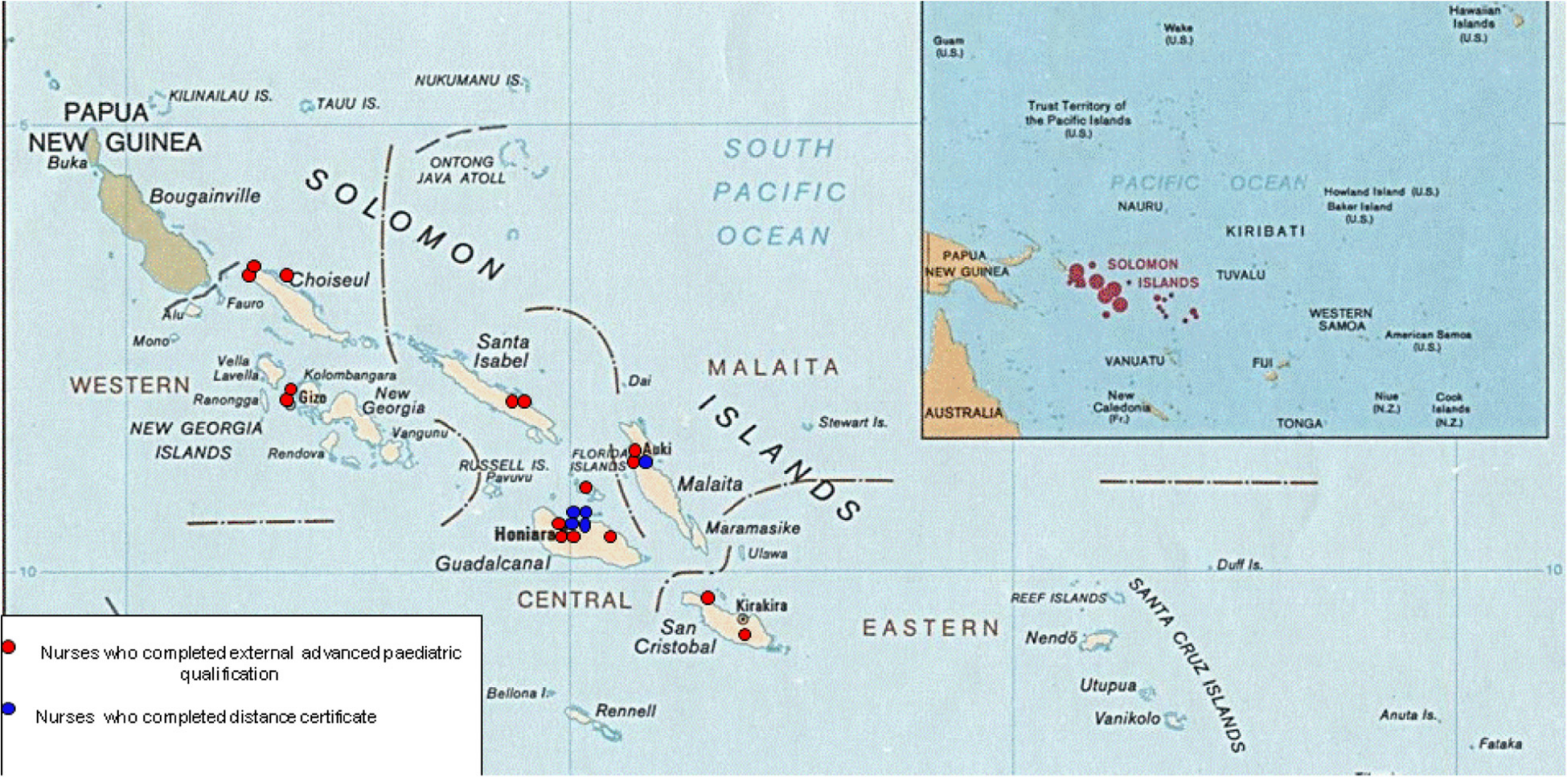
Distribution of nurses interviewed.

The nurses who had completed the post-basic paediatrics training through UPNG or an outside university were distributed throughout the country in hospitals and remote clinics. Those who undertook the distance course were concentrated in the main hospitals in Honiara, Western Province and Malaita province. There were no nurses with paediatric qualifications in two out of the nine provinces: Rennell-Bellona Province and Temotu Provine (Figure 1).

### Roles of nurses interviewed

All nurses were currently employed in teaching, clinical or management areas within the Solomon Islands Government health or nursing education sectors (Table 2), with the majority involved in clinical care. All 16 of the UPNG/New Zealand (NZ)-trained nurses interviewed were currently employed in senior roles as either nurses in charge of paediatric wards in the referral or provincial hospitals, sole clinicians working in remote and very remote clinics, in education delivery, or as provincial child health officers. Two of the nurses working at a provincial hospital had spent six months of 2010 with no doctor at their hospital or in the province. They ran the hospital including paediatric and midwifery wards with support available by telephone from Honiara.

**Table 2:** Employment location and roles of child health nurses in Solomon Islands

**Province**	**Location**	**Number**	**Roles of the nurses**
**Nurses who had completed a post-graduate diploma outside Solomon Islands**			
Choiseul province	Taro Hospital	2	Nurse 1: child health officer in province in charge of running MCH clinic at Taro, responsible for clinical care, community outreach, public health for example, collecting population-based data through the Family Health Card, and supervising junior staff.
			Nurse 2: nurse educator in charge of Choiseul province; coordinates all clinics and outreach services with supervision from Provincial Medical Director.
	Remote area health centre	1	Nurse 3: sole clinician working in remote clinic (> 4 hours by boat from nearest hospital); responsible for clinical paediatric and midwifery care, liaises daily via two-way radio with Taro hospital for support.
Western Province	Gizo Hospital	2	Nurse 4: clinical nurse in charge of children’s, midwifery and male wards; administrative and clinical supervisory duties.
			Nurse 5: clinical nurse instructor; supervises and teaches undergraduate nursing students on clinical rotation to children’s and midwifery wards.
Malaita Province	Kil'ufi Hospital	2	Nurse 6: clinical nurse in charge of children’s, midwifery and emergency wards; predominantly administrative and management role.
			Nurse 7: charge nurse on children’s ward; responsible for management of ward, staff rostering, supervising junior staff and clinical care.
Makira/Ullawa Province	Remote area health centre	1	Nurse 8: senior nurse at remote health centre, accessible only by boat (> 3 hours from provincial hospital). Responsible for clinical and public health care for paediatrics and midwifery and administration; liaises daily via two-way radio with Kirakira hospital for support.
Isabel Province	Buala Hospital	2	Nurse 9: senior nurse in charge of clinical areas, children’s ward, midwifery and medical wards; administrative, management and clinical duties.
			Nurse 10: clinical nurse responsible for children’s ward and midwifery.
Central Province	Tulaghi Hospital	1	Nurse 11: child health officer, responsible for child health for province, coordinates public health programme and outreach including EPI, clinical responsibilities in district hospital, supports junior nurses; clinic is 1 to 2 hours by boat to Honiara.
Guadalcanal Province	Grove Hospital	1	Nurse 12: senior nurse in charge of divisional hospital clinical areas; clinical and administrative duties, had worked for many years as sole clinician at a remote area health centre 4 to 6 hours by boat and road from Honiara.
Honiara	Children’s ward	1	Nurse 13: senior nurse in charge of children‘s ward, clinical, teaching, supervising, management and administrative duties; also provided clinical support to emergency department; had also completed distance course.
	Neonatal ward	1	Nurse 14: senior nurse in charge; administrative, clinical, management and supervising teaching duties.
	Ministry of Health nurse education unit	1	Nurse 15: education coordinator/nurse educator with senior teaching role; responsible for delivery of radio-based course for nurses in remote areas; planning, implementation and delivery of this curriculum.
	Solomon Islands College of Higher Education	1	Nurse 16: senior nurse lecturer; responsible for delivery of undergraduate nursing curriculum, examining of students and assisting with post-graduate course delivery.
**Nurses who had completed IPPC course by distance learning via University of Sydney**			
Honiara	National Referral Hospital Children’s ward	5	Nurse 17: staff nurse in children’s ward; clinical responsibilities in acute setting.
			Nurse 18: staff nurse in children’s ward; clinical responsibilities in acute setting.
			Nurse 19: staff nurse in children’s ward; clinical responsibilities in acute setting.
			Nurse 20: staff nurse in children’s ward; clinical responsibilities in acute setting.
			Nurse 21: emergency and outpatients nurse.
Malaita Province	Kilu'ufi Hospital	1	Nurse 22: clinical nurse consultant, senior nurse administrator; responsible for management and administration of children’s wards, emergency and midwifery and neonatal ward.

Four nurses were involved in major teaching roles; two at Solomon Islands College of Higher Education, training undergraduate nurses, and one at the Ministry of Health, providing distance education modules by radio to remote nurses. Two of these nurses had completed their paediatrics course in the late 1990s and were near retirement age. Another nurse was responsible for coordinating nursing and medical services for remote provinces as the Ministry of Health education coordinator. (Table 2).

Nurses in remote areas and provincial hospitals commonly provided radio support on a 24- hour basis to nurses working in very remote areas as well as undertaking outreach community visits to remote and very remote regions of their provinces.

### Nurses’ perceptions of their roles

A common theme was a feeling of being overwhelmed by the scope of their role without the support of similarly trained colleagues to rely on and either no doctors, or junior doctors with limited experience, to provide support and guidance. Nurses in clinical roles were commonly called upon after hours by other nurses with less experience, or by junior doctors, to triage and manage sick children.

‘We don’t have enough of the specialist nurses or doctors that can do it (triage and look after very sick children) so sometimes I have to come back 2 or 3 times at night to assess the baby, to put up this IV… The doctor if he is junior he will always call us to come back to manage the very sick child as he is not experienced and he wants our advice how to do it. But sometimes I just want to see my family but it is always me that gets called because I am the one with paediatric experience, there is no one else.’

‘For six months last year we had no doctors at all. I do the ward rounds, I do the admissions and discharges, the consultation with the Honiara doctors by telephone and they advise me to give this antibiotic or to send it (the sick child) over.’

‘I am a clinical nurse consultant I look after the paediatric ward, obstetric services and at the same time I am a midwife and at the same time anything that happens in the theatre I go to the theatre for caesarean sections and I take calls on the radio from nurses in the periphery when they need advice to treat.’

‘There as so many pikininis but too few nurses and doctors who know how to provide acute care so it all comes down to one or two of us and we can’t hope to do everything and there is no one coming on behind us to take over.’

Most nurses interviewed had administrative duties in addition to their clinical roles. These consisted of supervising wards, organising staff rosters, reporting on basic health statistics and completing reports to send to their supervisors or the Ministry of Health. When asked whether they received feedback all nurses said ‘no never’; when asked if feedback would be helpful they all stated that it would be.

A number of nurses felt that there was much emphasis on reproductive health with many midwives trained and working across the country, but little emphasis on supporting or training child health nurses to manage complex and difficult roles.

‘…the focus for many years from the Ministry has been on reproductive health and family planning and training more midwives, this is very important but there also needs to be support and focus on child health as these babies are not babies for so long…’

The most common difficulties the nurses interviewed faced were lack of similarly qualified colleagues. The majority of the nurses interviewed worked in remote or very remote locations, some with responsibility for a district or entire province.

### Nurses’ experience with external advanced paediatric courses

The initial interview questions examined the nature of each paediatric course attended by nurses, and its components. The University of Papua New Guinea course was seen by all nurses as being very beneficial to increase their skills in paediatrics.

‘Hem barava (it was good/fantastic) helpful for gud (good) especially in the rural clinic, hem (it was) really helpful because we have no doctors and nobody else there, so we just treat all childrens, only very serious cases we need to call the doctor (by two-way radio) and the other cases me pick hem up and treat hem pikinini quickly.’

‘I was general nurse when they sent me over to PNG so when I went there I learnt a lot from those people because before I don’t even know now to insert a drip in a child or manage a sick child. It relates very well to me to work in the remote hospital because now I know how to manage it all, it’s a good thing for me because when I see a sick child I know straight away that this child has something or something else and I know how to manage and treat this child myself now. It takes away the panic.’

Other comments related to the structure of the course and rotation between theoretical units and the university and clinical components undertaken in both the hospital and the community.

‘There was theory and clinical practice. We learned the theory and then we did the clinical practice and the competencies for assessment as well as theory exams. There was a lot of clinical practice, it was rotation on the wards as well as some community where we went out and spent eight weeks. We were supervised in the wards, there were a lot of doctors and nurses there that helped us learn and supervised us. We learnt a lot to bring back with us to help our practice in the Solomons. I was in the remote for a long time, that knowledge helped me a lot there as there were no doctors.’

Nurses were satisfied with the content of the UPNG paediatrics course, however all nine nurses who had undertaken the combined course in paediatrics and midwifery had comments about the difficulty in meeting all the clinical components and required competencies in the 10-month period available.

According to a paediatrician, Honiara, ‘It was very stressful for the nurses to have to complete both (paediatric and midwifery training) together in one year. So we stopped sending the nurses PNG in 2007 as it is very expensive and the course structure had changed for what we expected in just paediatrics. We need many more paediatric nurses, especially in the provinces… but we can only send a couple each year…so it would be better if we could start a course here, the midwifery training has been very successful.’

Many nurses expressed difficulty with separation from their family when they went to PNG for post-graduate studies.

‘When I went to PNG to do my paediatrics course, it was for one year and we couldn’t go home at all. I left my small baby and my husband and I couldn’t see them at all for nearly one year. That was the hardest thing.’

### Nurses experience with overseas distance education

The distance education course (International Post graduate Paediatric Course- IPPC, through the University of Sydney) [9] was perceived by the nurses to be interesting but of limited relevance to their practice in the Solomon Islands, and far advanced in terms of the level of material presented and mode of delivery. The nurses were also constrained by lack of technology, computers or internet access to view the internet material or play the DVDs. The course was first offered in the Solomon Islands in 2009, and was delivered at three main centres in the Solomon Islands: NRH in Honiara, Gizo Hospital Western Province, and Kilu’ufi Hospital in Malaita province. All nurses interviewed were concerned by the lack of clinical supervision and mentoring. The difficulties faced by the nurses undertaking the IPPC were increased when the course was delivered outside Honiara.

One nurse said: ‘It is difficult for us to do it here (IPPC Course) we just need the support and some answers for all the practicals, some supervision and resources, sometimes with this course you feel neglected, because at the hospital you don’t have any time and no mentors and no practical. We have some questions but the doctors are too busy with the wards most of the time and so there is no support either to do his work and help us with this course. It is very hard for all or us and none of us pass this course.’ Four out of the five nurses interviewed said they thought the course was actually aimed at doctors. The nurses stated that they found it interesting, but unhelpful to learn about equipment and resources available in Australia and ‘the luxury setting’ but not in the Solomon Islands: ‘I think that the thing I want to say it that they have to look at our setting, because in the course like the treatments they have ….what can I say they just have a different level, there in Australia. Because in the Solomon Islands we have still different treatments and antibiotics. Some antibiotics that they’re included in the treatments of the paediatric kids out there, we don’t have it here. Some is appropriate, but they need to see that the management is in line with what we have and in our setting with the equipments and things like that it is all very different than our setting.”

## Discussion

Child illness and deaths remain high in the Solomon Islands, as in many Pacific countries, and the number of health workers is inadequate to provide the services required. A comprehensive child health programme that can further reduce child mortality and address significant morbidity issues has many components; acute clinical care, immunisation, nutrition, public health, coordination of care for chronically unwell children. Each of these components is increasingly complex, with the introduction of new treatments, increasing antimicrobial resistance, emerging disease threats, and the recognition of previously neglected areas (such as neonatal care, adolescent health, child protection and disability). With these demands, and the reality that there will not be sufficient doctors to address these needs in remote areas in the Pacific, there is a need to equip nurses with the skills required in these areas.

The issue of child health nurses worldwide has received almost no attention, despite the global push to improve child survival. There have been few previous studies examining the roles of child health nurses in developing countries, and we could find only one from Lesotho [10]. A few studies have focused on training nurses in narrower speciality roles, such as paediatric oncology [11], neonatal resuscitation [12], or the advanced paediatric life support programme [13]. Invariably these studies have focused on the contribution of nurses or doctors from Western countries to the training of nurses in developing countries [14]. There is no previous study to have evaluated the long-term output and outcomes of local child health nursing education in a developing region.

This study provides evidence that nurses can fulfil the roles required by a provincial health service in a developing country in the 21^st^ century, long after they have completed training. In the Solomon Islands, child health and paediatric nurses were employed in vital clinical, public health, management and teaching roles. Many worked as sole practitioners in remote provincial settings where there were no doctors. Most were called upon to advise and assist junior doctors as they were the clinicians with the most experience in child health at their hospital or health centre. This has implications for paediatric care across the country, and creates opportunities for an appropriate approach to the human resource of countries such as the Solomon Islands in this time, towards and beyond the MDGs.

Overall, the nurses gave positive feedback about the quality of training in the 12-month residential diploma in paediatrics, or paediatrics and midwifery through UPNG. A recurrent theme was relevance; that such courses, with their emphasis on clinical competency, mentorship and supervision, equipped nurses to function independently, particularly in remote, isolated settings. However, the PNG paediatrics post-graduate course, and support required to undertake it, is expensive at approximately (SI $180,000 per nurse per year). There is also a drain of skilled staff, albeit temporarily, and overseas training results in the removal of the nurse from the local workforce for 12 months. Most concerning for nurses involved in overseas training, was the separation from their families and communities. No Solomon Island nurses have been trained at the University of PNG since 2006 for reasons of cost, social dislocation and concern about the combined midwifery and child health course not providing a sufficient level of competency. UPNG has now reverted to single midwifery and child health programmes because of concerns that the combined course did not allow the students to obtain the required competency level in the time provided, and these reforms have been positively received. However there is now only one post-basic child health nursing course in PNG, where formerly there were four. The advocacy for midwifery training, appropriate given the high maternal mortality in the Pacific, has overshadowed the need for training more child health nurses and the need for re-establishing colleges who can train such staff. Redressing of this imbalance is urgently needed.

The nurses in this study who had had undertaken a distance education certificate identified as challenges, the lack of supervised clinical training, difficulties in accessing the technology, and the inappropriateness of the content and delivery method to the local setting.

Other local initiatives have attempted to address the lack of training opportunities for nurses, including a programme for training provincial nurses on the management of seriously ill children using the WHO Pocketbook of Hospital Care for Children. Two hundred nurses in all provinces have been trained in the use of these guidelines [15]. The Integrated management of Childhood Illness (IMCI) training was also conducted in selected provinces, but has not been scaled up nationally [16]. Interviewed nurses concluded that there was a need for in-country post-graduate training for nurses in child health. The current numbers of post-graduate child health nurses provide inadequate and fragmented cover.

This study has demonstrated the need for advanced paediatric trained nurses in the Solomon Islands, and its value in equipping nurses who work in remote areas with capacity to fulfil the roles required of them. Current modes of delivery have not been able to fulfil this need according to the nurses interviewed. More than 120 graduates from the Solomon Islands post-graduate midwifery course, facilitated jointly by the Ministry of Health and Solomon Islands College of Higher Education for over 15 years, are now widely distributed throughout the Solomon Islands. This has been a substantial success. This success could be replicated with a locally delivered, content-appropriate nursing course in paediatrics and child health. The content of such a course would be based on the child health needs of the Solomon Islands, and draw upon the many training resources and standards from WHO, United Nations Children’s (Emergency) Fund (UNICEF) and other agencies in recent years [15-18], and would also draw upon the UPNG course.

There are resource implications. However many of the ingredients are in place and this type of course can be relatively low budget yet still of high quality. Such a course is ideally practically based, therefore, much of the training would be conducted in the paediatric ward at the NRH. This requires input from clinical teachers and paediatricians; however, there is a benefit to quality of care even while the students are training. This is because of the increased focus on clinical teaching, and the level of critical enquiry that ensues from becoming a teaching institution. There are classrooms at most major hospitals for theoretical teaching, so infrastructure requirements are minimal. The public health teaching required for such a course is largely practical, and requires involvement of public health branches of the health department, both national and provincial, plus travel to rural areas for practical experience of public child health programs. Clinical nurse teachers need higher training, and most nursing colleges in the Pacific require teachers and lecturers to be trained to Master’s level in nursing or education. This could be facilitated in collaboration with universities in Australia. However even if provided for only two or three nurse teachers (the number required to establish a course training 15 to 20 paediatric nurses per year), this would require substantial funding. Higher degree education in Australia is expensive at approximately AU$25,000 for a Master’s degree, plus travel and living expenses, which can cost an additional $40,000 a year. The graduates of a paediatric nursing course would require professional and legal recognition and accreditation for a wider scope of practice. There are precedents in midwifery, but changing accreditation and legal recognition requires political will and action, as well as modest but ongoing funding for higher wages for specialist nurses.

## Conclusions

A local paediatric nursing course in the Solomon Islands would take candidates from all provinces, and would build strong knowledge and skills for child health throughout the country, and develop a professional network for leadership and advocacy for child health.

## Competing interests

TD has an honorary appointment at the School of Medicine in University of PNG where one of post-graduate paediatric nursing course is offered. The Centre for International Child Health and TN have been actively involved in training for nurses on the WHO Pocket Book of Hospital Care for Children in Solomon Islands. No authors have any financial conflict of interest with the themes or issues presented in this paper.

## Authors’ contributions

SC, TD, RS and DO designed the study. SC and MT were involved in the interviews and data collection. SC and TD wrote the paper, and all authors had inputs into various versions. All authors read and approved the final manuscript.
